# Work–life conflict, coaching, and workplace harassment as determinants of employee well-being

**DOI:** 10.1038/s41598-026-51240-4

**Published:** 2026-04-30

**Authors:** Eun-Mi Baek, Hyeon Jo

**Affiliations:** 1 Department of Preventive Medicine, College of Medicine, Catholic University of Korea, 222 Banpo-daero, Seocho-gu, Seoul, 06591 Republic of Korea; 2 Present Address: Headquarters, HJ Institute of Technology and Management, Seoul, Republic of Korea; 3 Kookmin Information Technology Research Institute, Kookmin University, Seoul, Republic of Korea

**Keywords:** Workplace dynamics, Employee well-being, Engagement, Work-life conflict, Sexual harassment, Psychology, Health occupations

## Abstract

In today’s complex workplace environments, understanding the drivers of employee engagement and well-being is essential. This study draws on data from the 6th Korean Working Conditions Survey (*N* = 33,063) and applies Partial Least Squares Structural Equation Modeling (PLS-SEM) to examine how key workplace dynamics shape these outcomes. The analysis focuses on the effects of work-life conflict, life-work conflict, supervisory coaching, gender diversity, and personal life factors. Results confirm the negative impact of work-life conflict on both engagement and well-being, while life-work conflict, although reducing engagement, unexpectedly enhances subjective well-being by providing meaning through personal responsibilities. Supervisory coaching emerged as a strong positive driver of both outcomes, and the proportion of women in the workplace was linked to improved well-being. Household size also showed a dual role, increasing conflict but contributing positively to well-being. These findings highlight the dual-edged nature of conflict and the resource-providing role of supportive leadership and family responsibilities, offering valuable insights for organizational policies and interventions.

**Supplementary Information:**

The online version contains supplementary material available at 10.1038/s41598-026-51240-4.

## Introduction

In recent years, the modern workforce has experienced transformative shifts in employees’ work–life dynamics^1^. As societal norms evolve, there is increasing scholarly and policy interest in achieving work–life balance, which is now recognized as a key factor in promoting both employee well-being and organizational success^2,3^. Governments and organizations worldwide are implementing policies to support this balance. For example, the European Union’s ‘Work-Life Balance Directive’ aims to introduce new rights for parents and caregivers, fostering a better distribution of caring responsibilities between men and women^4^. Additionally, many multinational corporations have begun to offer flexible working hours, work-from-home options, and employee wellness programs to accommodate the diverse needs of their workforce^5^. These developments reflect evolving societal expectations regarding the integration of work and personal life, where contemporary employees are increasingly advocating for a more integrated and harmonious blend of their professional and personal spheres. This shift has made the study of work engagement and subjective well-being a necessity, considering their critical roles in the overall health and productivity of the workforce^6–9^. Recent research shows that work engagement itself can serve as a mediating pathway between work–family balance and well-being, highlighting its role in reducing turnover intentions and strengthening positive organizational attitudes^10^.

The investigation of work-life balance is crucial, especially in an era where the lines between work and personal life are increasingly blurred^11^. Work-life balance is not just about managing time between work and personal activities, but also about minimizing conflict between these two domains^12–14^. Specifically, this study delves into the impacts of work-life conflict and life-work conflict separately, acknowledging that challenges in one area can significantly affect the other. Work-life conflict occurs when job demands hinder personal life, while life-work conflict happens when personal responsibilities impede work performance. Exploring these distinct aspects is vital for understanding the full spectrum of work-life balance and its effects on individuals.

The impact of supervisory coaching on fostering work engagement and enhancing subjective well-being is profound and multifaceted. When supervisors engage in effective coaching, it not only cultivates a supportive work environment but also significantly boosts employee motivation and job satisfaction^15,16^. Various organizations exemplify this through leadership development programs that emphasize coaching skills^17^. For instance, companies like Google and IBM have implemented coaching cultures where supervisors are trained to provide constructive feedback, set realistic goals, and offer emotional support, thereby enhancing overall employee performance and well-being^18^. This study also delves into the influence of supervisor gender on coaching effectiveness. Research suggests that male and female supervisors may adopt differing coaching approaches, with potential variations in their impacts on employee outcomes^19,20^. For instance, female supervisors might lean more towards a transformational leadership style, which has been associated with high levels of employee engagement and satisfaction^21^. Understanding these gender-based differences is crucial for tailoring leadership strategies to meet the diverse needs of today’s workforce.

Furthermore, the effect of household size on work-life balance presents a unique area of exploration. Household responsibilities, often unequally distributed between genders, can significantly influence an employee’s ability to manage work-life balance. Women, in many contexts, tend to be more affected by household size than men, potentially leading to greater life-work conflict. Investigating these effects across genders is necessary to comprehend fully how household dynamics intersect with professional life^22,23^. This aligns with prior findings indicating that psychological capital and its link to well-being are strongly moderated by gender, suggesting that women and men may interpret and experience resource gains and losses differently in relation to household responsibilities^24^.

Gender diversity in the workplace plays a crucial role in shaping both employee engagement and subjective well-being^25^. Diverse gender representation brings varied perspectives and experiences, fostering a more inclusive and innovative work environment^26^. Studies have shown that organizations with higher gender diversity tend to have more engaged employees, as diversity can enhance creativity and problem-solving abilities^27^. For instance, companies like Accenture^28^ and Salesforce^29^ have actively promoted gender diversity and reported improvements in employee morale and productivity. Moreover, gender diversity has been linked to higher job satisfaction and well-being, as employees feel more valued and included in such environments^30^. Beyond organizational outcomes, gender inclusivity has also been conceptualized as part of broader sustainable development and social inclusion goals, framing workplace well-being within a global sustainability discourse^31^.

Sexual harassment and violence in the workplace are critical issues that have a profound impact on employees’ subjective well-being^32–34^. These negative experiences can range from inappropriate comments and unwanted advances to physical assaults, creating an environment of fear and distress. For example, a scenario where an employee is subjected to unwelcome sexual remarks or intimidation by a colleague or supervisor not only undermines their sense of safety but also their mental health and job satisfaction^35,36^. Moreover, Yesildag et al.^37^ show that a strong violence-prevention climate in healthcare is significantly associated with higher workplace happiness, emphasizing the role of organizational safety and cultural context in shaping well-being. Considering both sexual harassment and workplace violence in explaining employee well-being is essential because they directly affect psychological safety, a key component of employee well-being. The presence of such negative behaviors leads to increased stress, anxiety, and a decline in morale, all of which are detrimental to the overall mental health and productivity of employees^38^.

Despite this growing scholarly attention, several gaps remain. First, many prior studies have not clearly distinguished between work–life conflict and life–work conflict, often treating them as a single construct despite their different antecedents and consequences. Second, supervisory coaching, gendered workplace structures, household responsibilities, and exposure to harassment or violence have typically been examined in isolation rather than within an integrated framework. Third, most evidence comes from Western contexts, leaving limited understanding of how these dynamics operate in collectivist and hierarchical work cultures such as South Korea. These gaps highlight the need for a multidimensional, context-sensitive approach, which the present study addresses.

This study is grounded in two complementary theoretical frameworks: the Job Demands–Resources (JD-R) model and Conservation of Resources (COR) theory. The JD-R model explains how job demands, such as overload, conflict, or harassment, deplete energy and impair well-being, while job resources, including recognition, fairness, and supervisory coaching, foster engagement and motivation^39,40^. COR theory further posits that individuals strive to acquire and preserve valuable resources, and that resource loss leads to stress and diminished well-being, whereas resource gain promotes resilience and satisfaction^41,42^. Integrating these frameworks provides a robust lens for understanding how both demands and resources interact to influence employee engagement and well-being. This theoretical framing is consistent with recent work showing that supervisory support and work attitudes (e.g., job satisfaction, engagement) act as critical mediators between work–family balance and workplace well-being, reinforcing the multidimensional approach adopted here^43^. By applying these theories to a multidimensional model that distinguishes between work-life and life-work conflict, incorporates supervisor characteristics, and considers household and organizational contexts, this study contributes to advancing theory-driven explanations of workplace dynamics.

Previous research has widely examined work-life conflict^44–46^, supervisory support^47,48^, gender diversity^25,49,50^, and harassment^33,51,52^, yet most studies have treated these themes in isolation, limiting their explanatory power. This study contributes by adopting a multidimensional perspective that integrates personal, relational, and organizational factors to capture the complex determinants of engagement and well-being. A central originality lies in distinguishing between work-life conflict and life-work conflict, which are often conflated in prior literature^53–55^, despite their differing mechanisms and outcomes. This separation enables a more refined analysis of how demands from work versus personal life uniquely affect employees. In addition, this study examines underexplored intersections, such as how household size shapes well-being and how supervisor gender influences coaching and engagement, extending beyond traditional leadership research. While coaching, fairness, and diversity have been studied extensively, their collective interplay with harassment and violence in shaping subjective well-being has rarely been empirically tested on large-scale data. Drawing from the 6th Korean Working Conditions Survey with 33,063 participants, the purpose of this study is to clarify these multifaceted relationships, advance theoretical understanding, and offer practical insights for workplace policies that strengthen employee well-being.

## Theoretical framework and hypothesis development

**Fig. 1:**
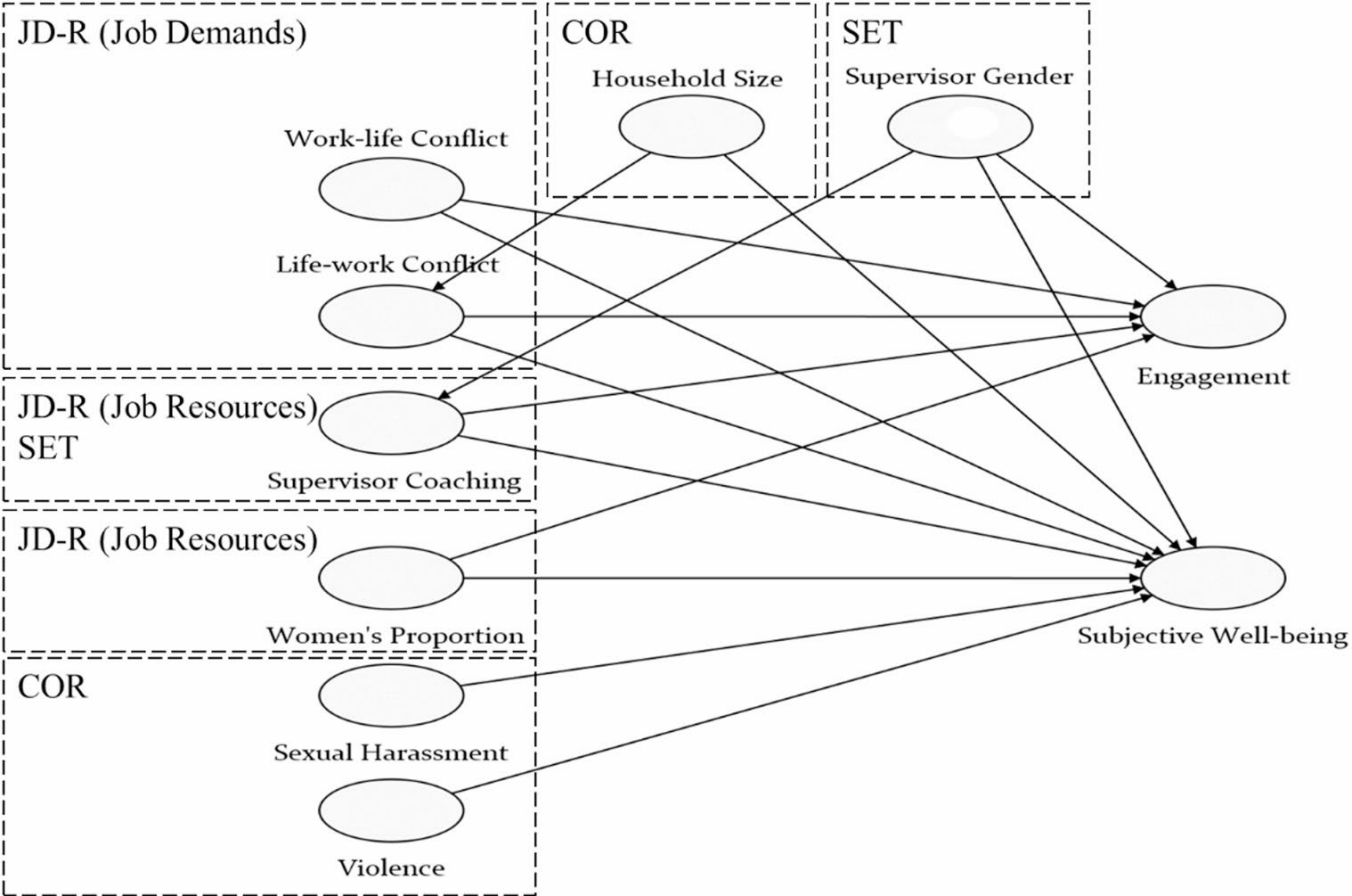
Research Model. SET stands for SET.

The theoretical framework of this study is anchored around core theories that provide a robust foundation for understanding the interrelationships among various workplace factors and their impact on employee engagement and subjective well-being. These core theories include the JD-R model, the COR theory, and social exchange theory (SET), which together offer a comprehensive perspective on the dynamics at play in organizational settings. The JD-R model^39^ forms the primary theoretical underpinning of this research, explaining how various workplace factors function either as demands or resources. This model posits that while job demands (like work-life conflict and life-work conflict) can lead to strain and are associated with costs for employee well-being, job resources (such as supervisory coaching and supportive workplace environments indicated by women’s proportion) can buffer the impact of job demands and are instrumental in fostering employee engagement and enhancing well-being. COR theory^41^ complements the JD-R model by emphasizing the importance of resources in maintaining and protecting one’s well-being. This theory suggests that the threat of resource loss, actual loss, or lack of adequate resource gain following resource investment causes stress. COR theory is particularly relevant in understanding how factors like household size and the effects of sexual harassment and workplace violence can impact employees’ resource pools and consequently their psychological outcomes. SET^56^ is utilized to delve into the dynamics of supervisor-subordinate relationships and their consequences on organizational loyalty, commitment, and satisfaction. It provides a framework for understanding how supervisory coaching, influenced by supervisor gender, constitutes a relational transaction that can lead to positive reciprocal actions from employees, such as increased engagement and improved well-being. By integrating these theories, the study offers a holistic view that not only addresses the direct impacts of specific workplace factors on employee outcomes but also captures the complex interplay between these elements. The JD-R model and COR theory together highlight how the management of workplace demands and resources is crucial for organizational success, while SET underscores the significance of interpersonal relationships and social interactions in the workplace. Figure 1 presents the research model for this study, which comprehensively examines the dynamics between multiple workplace factors.

### Work-life conflict

Work-life conflict occurs when the demands of work and personal life are mutually incompatible, leading to a strain in one or both domains^57^. This concept has been extensively studied in the context of its impact on various aspects of employee well-being and performance^44–46^. Regarding engagement, a key aspect of employee performance, past research has consistently shown that work-life conflict can lead to decreased levels of engagement^58–60^. This is primarily because the stress and exhaustion stemming from juggling work and personal responsibilities can diminish one’s energy and enthusiasm towards work^61^. Employees experiencing high levels of work-life conflict often report feeling drained and unable to fully immerse themselves in their work tasks, leading to lower engagement levels^62^. Similarly, the influence of work-life conflict on subjective well-being has been a focal point in occupational health psychology. Subjective well-being, which encompasses an individual’s overall satisfaction with life, positive emotions, and low levels of negative emotions, is significantly impacted by work-life conflict. Research indicates that when employees struggle to balance work and life demands, it not only causes stress and anxiety but also impairs their overall life satisfaction and emotional well-being^63^. This negative impact is particularly pronounced when work demands encroach on family time or personal activities, leading to a reduction in subjective well-being^64^. In line with the JD-R model, work-life conflict represents a demand that drains employees’ energy and hinders their engagement, while the COR theory highlights how the persistent loss of time and energy resources undermines well-being. These theoretical perspectives directly frame the hypotheses by showing how incompatible work and life demands operate as resource-depleting stressors. Thus, this study suggests the following hypotheses:

#### H1a

Work-life conflict is expected to negatively influence engagement.

#### H1b

Work-life conflict is expected to negatively influence subjective well-being.

### Life-work conflict

Life-work conflict, a concept distinct yet related to work-life conflict, focuses on the interference of personal life with work responsibilities^2,65^. This phenomenon occurs when the demands of one’s personal life hinder the ability to fulfill work-related duties effectively^66,67^. In the context of employee engagement, life-work conflict has been shown to have a detrimental impact. The strain and preoccupations of personal responsibilities often lead to reduced cognitive and emotional resources available for work, thereby decreasing engagement levels. Research by Halbesleben^68^ indicates that employees struggling with life-work conflict are likely to experience a decline in their enthusiasm and dedication at work, ultimately affecting their overall engagement. Concerning the effect on subjective well-being, life-work conflict poses a significant threat. The continuous struggle to balance personal responsibilities with work demands can lead to heightened stress levels and reduced overall life satisfaction, an essential component of subjective well-being. According to the JD-R model, life-work conflict represents a demand that drains essential personal resources before they can be invested in work, undermining engagement. From the COR perspective, the persistent spillover of personal demands into work contexts accelerates resource loss, thereby reducing overall well-being. These theoretical foundations clarify how life-work conflict undermines both work-related outcomes and general life satisfaction. Thus, this study suggests the following hypotheses:

#### H2a

Life-work conflict is expected to negatively influence engagement.

#### H2b

 Life-work conflict is expected to negatively influence subjective well-being.

### Supervisory coaching

Supervisory coaching refers to the process where supervisors engage in behaviors that promote learning, development, and performance improvement among their subordinates^69^. The impact of supervisory coaching on employee engagement is profound. Engagement is significantly boosted when employees receive supportive coaching from their supervisors^16^. Studies have shown that when supervisors provide constructive feedback, guidance, and support, it enhances employees’ connection to their work, thereby boosting their engagement levels^70,71^. Similarly, the role of supervisory coaching in enhancing subjective well-being is substantial. Subjective well-being can benefit from the supportive and developmental nature of coaching. Supervisory coaching contributes to a positive work environment, where employees feel valued, supported, and understood. This positive work environment, in turn, fosters higher levels of subjective well-being by reducing work-related stress and improving job satisfaction^72^. The personal attention and tailored guidance that coaching entails can significantly enhance an employee’s emotional well-being, leading to greater overall life satisfaction^47^. According to the JD-R model, supervisory coaching represents a vital job resource that replenishes employee energy, counterbalances job demands, and stimulates engagement. From the perspective of SET, coaching fosters reciprocal trust and commitment, enhancing employees’ motivation and well-being. These theoretical lenses clarify why supervisory coaching strongly predicts both engagement and subjective well-being. Thus, this study suggests the following hypotheses:

#### H3a

Supervisory coaching is expected to positively influence engagement.

#### H3b

Supervisory coaching is expected to positively influence subjective well-being.

### Women’s proportion

Women’s proportion, in this study, refers to the proportion of women within a workplace or organizational setting. The influence of gender diversity on employee engagement is a topic of growing interest, particularly in organization research^50,73^. Prior research demonstrates that gender composition can shape the quality of work environments, influencing both relational and psychological outcomes^74–76^. A higher proportion of women has been shown to foster inclusivity and collaboration, which supports greater engagement among employees^77,78^. Moreover, women’s presence in diverse organizational roles can disrupt traditional hierarchies and stimulate a more dynamic and engaging culture^79–82^. With regard to subjective well-being, research highlights that balanced gender representation is associated with reduced stress, enhanced job satisfaction, and a stronger sense of belonging^83–85^. Gender-diverse teams also show improved communication and collaboration, which contribute positively to well-being^86,87^. Integrating these findings, this study positions women’s proportion within the JD-R model as a resource that fosters engagement and well-being^88^, and within COR theory as a contextual support that protects psychological resources against depletion. Thus, this study suggests the following hypotheses:

#### H4a

Women’s proportion is expected to influence engagement.

#### H4b

Women’s proportion is expected to influence subjective well-being.

### Household size

Household size, a demographic factor, refers to the number of individuals living in a single household. Its impact on life-work conflict is quite significant, as life-work conflict involves the challenges and strains arising from personal or home responsibilities affecting work performance^89^. Larger household sizes often imply greater domestic responsibilities and potential caregiving demands, which can encroach upon work commitments, leading to increased life-work conflict^90^. This relationship is supported by findings that indicate an escalation in life-work conflict with an increase in household size due to the additional time and energy required for household duties and caregiving, leaving less capacity for work-related tasks^91^. However, the influence of household size on subjective well-being presents a more complex picture. While larger households might imply more responsibilities and potential stressors^92^, they can also provide a supportive and enriching environment, contributing positively to an individual’s sense of belonging, emotional support, and overall life satisfaction^93,94^. Research has shown that the social support and companionship provided by family can buffer against stress and enhance subjective well-being, despite the increased responsibilities^95^. The COR theory provides a foundation here, suggesting that larger households may simultaneously increase resource loss through heightened demands while also offering resource gains through emotional support. In parallel, the JD-R model explains how household-related demands create additional life-work conflict, yet resources embedded in family support can enhance well-being. Thus, this study suggests the following hypothesis:

#### H5a

Household size is expected to positively influence life-work conflict.

#### H5b

Household size is expected to positively influence subjective well-being.

### Supervisor gender

Supervisor gender, a key variable in organizational studies, relates to whether a supervisor is male or female in a workplace setting^96^. The influence of supervisor gender on supervisory coaching is a subject of considerable interest. Supervisory coaching, which involves guiding and supporting subordinates for performance improvement, can vary based on the gender of the supervisor^15^. Research indicates that female supervisors tend to adopt a more transformational style of leadership, which includes aspects of coaching and mentorship, compared to their male counterparts^21,97,98^. This orientation can lead to stronger supervisory coaching because subordinates perceive higher levels of guidance and developmental feedback^15^. In terms of engagement, male supervisors often emphasize transactional leadership and task-orientation, which may foster efficiency but can reduce relational quality, thereby lowering employees’ enthusiasm and involvement^99,100^. Finally, differences in leadership style also extend to subjective well-being, as female supervisors tend to promote inclusive climates that reduce stress and enhance employee satisfaction, whereas male supervisors may be perceived as more hierarchical, potentially limiting psychological safety^101,102^. From the lens of SET, gender-specific leadership approaches shape the reciprocity of trust and support between supervisors and subordinates, while the JD-R model conceptualizes supervisor gender as shaping the effectiveness of leadership as a job resource. Thus, this study suggests the following hypotheses:

#### H6a

Supervisor gender is expected to influence supervisory coaching.

#### H6b

Supervisor gender is expected to influence engagement.

#### H6c

Supervisor gender is expected to influence subjective well-being.

### Sexual harassment

Sexual harassment, a serious issue in the workplace, is defined as unwelcome sexual advances, requests for sexual favors, and other verbal or physical conduct of a sexual nature^103^. Victims of sexual harassment often report a range of negative psychological outcomes, including increased stress, anxiety, depression, and diminished life satisfaction^38,104^. These psychological impacts are not just momentary; they can have long-lasting effects on the overall emotional and mental health of the individuals involved^105^. Furthermore, the pervasive and insidious nature of sexual harassment can create a hostile and stressful work environment, contributing to a decrease in overall well-being^32,34^. The COR theory provides the basis for this hypothesis by suggesting that individuals strive to retain and protect personal and psychological resources, and when harassment erodes these resources, subjective well-being declines^41^. In addition, the JD-R model conceptualizes sexual harassment as a severe job demand that not only depletes emotional energy but also impairs health and well-being^39^. Thus, this study suggests the following hypothesis:

#### H7

Sexual harassment is expected to negatively influence subjective well-being.

### Violence

Violence in the workplace, encompassing physical assaults, threats, bullying, and other forms of aggressive behavior, is a critical concern in occupational health^106^. The detrimental impact of such violence on subjective well-being, which includes elements like life satisfaction, emotional balance, and overall mental health, is substantial. Experiences of violence at work can lead to a range of negative psychological outcomes, including increased stress, anxiety, depression, and a general decrease in life satisfaction^107^. These psychological effects are profound, often persisting long after the violent incidents, and can deeply affect an individual’s sense of safety and well-being. Moreover, the stress and trauma associated with workplace violence can lead to a pervasive sense of vulnerability, adversely affecting employees’ mental health and their ability to enjoy and find satisfaction in both their personal and professional lives^108,109^. From a theoretical perspective, the COR theory posits that violent experiences erode critical personal and psychological resources, directly undermining subjective well-being^41,110,111^. Similarly, the JD-R model conceptualizes workplace violence as an extreme job demand that drains emotional energy and disrupts recovery, ultimately leading to lower well-being^39,112,113^. Thus, this study suggests the following hypothesis:

#### H8

Violence is expected to negatively influence subjective well-being.

## Empirical methodology

Since this study involved the analysis of secondary data, it was exempt from review. Exemption from review was obtained from the Public Institutional Review Board Designated by Ministry of Health and Welfare (IRB) (Exemption No: P01-202404-01-035).

### Measurement instruments

This study utilized secondary data from the 6th Korean Working Conditions Survey (KWCS), conducted between 2020 and 2021. The KWCS is a nationally representative dataset that collects detailed information on employment conditions, psychosocial risks, and well-being among workers in South Korea. The dataset is publicly available and can be freely accessed and downloaded from the official KOSHA data portal: https://oshri.kosha.or.kr/eoshri/resources/KWCSDownload.do Because KWCS is a large-scale national survey designed primarily for public policy rather than academic research, it does not report original Cronbach’s alpha values for each construct, nor does it cite academic sources for item development. Accordingly, we employed the KWCS items directly, while presenting related academic literature to demonstrate the conceptual legitimacy of each scale. Where possible, three representative items are reported in parentheses as examples. For constructs in which wording or emphasis differed from existing validated scales, all relevant items are presented to ensure transparency.

Work-life conflict was measured using three items on a 5-point Likert scale (1 = very infrequently, 5 = very frequently): (1) “I keep worrying about work even when I’m not working,” (2) “I am so tired after work that I can’t complete the housework I need to do,” and (3) “I cannot dedicate as much time to my family as I would like because of work.” Similar concepts have been investigated in previous studies^114,115^.

Life-work conflict was measured with two items on a 5-point Likert scale: (1) “It is difficult to concentrate on work because of housework” and (2) “I can’t spend enough time on my work because of housework.” These items reflect established operationalizations of family-to-work conflict^2,65^.

Supervisory coaching was assessed with three items on a 5-point Likert scale (1 = strongly disagree, 5 = strongly agree), asking whether the immediate supervisor: (1) “is helpful in handling the work,” (2) “gives useful advice (feedback) about the work,” and (3) “encourages and helps me to develop.” This aligns with coaching measures in prior work^70,116^.

Women’s workplace proportion was measured with a single categorical item: “What is the approximate percentage of women among the employees at your current place of employment?” Responses ranged from 1 (none or almost none) to 5 (all or almost all). Household size was measured with a single demographic item: “How many individuals live in your household?”. Supervisor gender was captured with one item: “Is your immediate supervisor (direct superior) male or female?”

Engagement was measured with three items on a 5-point frequency scale: (1) “I feel full of energy when I work,” (2) “I am passionate in my work,” and (3) “Time flies when I work.” These correspond to engagement constructs^117,118^.

Subjective well-being was measured with three items on a 6-point frequency scale: (1) “I feel calm and comfortable,” (2) “I am active and energetic,” and (3) “My daily life is full of interesting things.” These items are consistent with prior well-being concepts^119,120^.

Sexual harassment was assessed with two items asking whether the respondent had experienced in the past month: (1) “unwanted sexual attention” or (2) “sexual harassment.” Violence was measured with five items assessing experiences in the past month: (1) “verbal abuse,” (2) “threats,” (3) “offensive behavior,” (4) “physical violence,” and (5) “bullying/harassment.” These align with variable concepts of workplace violence^121,122^.

To maintain comparability, Likert scales were recoded where necessary so that higher values reflected more positive or frequent experiences. This approach ensured conceptual consistency while leveraging the nationally representative scope of the KWCS data.

### Subject and data collection

This study is based on data collected from the 6th Korean KWCS, which targeted employed individuals aged 15 and above across South Korea. The survey was comprehensive in scope, covering employees from 50,000 households, with one eligible household member participating from each. The geographic scope of the survey spanned all 17 cities and provinces in the country. The target population for this survey included all household members aged 15 or older who were employed at the time of the survey. This encompassed workers, business owners, and self-employed individuals residing in South Korea. The survey sample was drawn from households within the census enumeration districts registered by the Statistics Korea as of 2018. Systematic sampling was utilized, with a probability-proportional-to-size systematic sampling for the primary sampling units (PSU), systematic sampling within each enumeration district for the secondary sampling units (SSU), and the selection of one eligible household member for the final survey unit (FSU). In cases where multiple eligible individuals were present in a household, a tablet program randomly selected the respondent. The data collection method employed was a one-on-one personal interview conducted by professional interviewers through household visits. The Tablet PC Assisted Personal Interviewing (TAPI) technique was utilized for this purpose. The survey period spanned approximately 22 weeks, from October 5, 2020, to April 11, 2021.

Table 1 presents the demographic characteristics of the study’s sample, which includes 33,063 participants. In terms of employment type, the survey distinguished between regular workers, who are either unrestricted by employment contract duration or have a contract lasting more than one year (or are not bound by a formal employment contract but are subject to standard recruitment procedures, HR policies, and eligible for severance pay), and temporary workers, who are employed for a period ranging from more than one month to less than one year, typically for specific projects or tasks with a duration of less than one year.

**Table 1:** Demographic Characteristics of the Samples.

Item	Subjects (*N* = 33,063)	Frequency	Percentage
Gender	Male	15,503	46.9%
	Female	17,560	53.1%
Age	15–19	147	0.4%
	20–29	4285	13.0%
	30–39	7013	21.2%
	40–49	7897	23.9%
	50–59	7545	22.8%
	60–69	3931	11.9%
	70–79	1676	5.1%
	80–89	556	1.7%
	> 90	13	0.0%
Employment type	Regular Employees	25,285	76.5%
	Temporary Employees	5855	17.7%
	Daily Employees	1923	5.8%

## Analysis and results

The employment of PLS-SEM in this study is justified by its ability to analyze complex models with multiple constructs, particularly suitable for exploratory research where theory is still developing^123^. PLS-SEM is advantageous in handling large and complex datasets, as in this study with a substantial sample size of 33,063 participants. It effectively deals with issues of multicollinearity and is less stringent about normality assumptions, making it appropriate for the diverse and non-normally distributed variables in this survey^124^. This approach also facilitates the examination of relationships between latent variables, crucial in uncovering nuanced insights in workplace dynamics and employee well-being^125^.

### Measurement model

The measurement model for this study was meticulously validated using several statistical methods to ensure the reliability and validity of the constructs. Table 2 outlines the scale reliability and validity, highlighting the Cronbach’s Alpha, Composite Reliability (CR), and Average Variance Extracted (AVE) for each construct. The constructs, including work-life conflict, life-work conflict, supervisory coaching, and engagement, demonstrated strong factor loadings and high levels of reliability and convergent validity. For instance, work-life conflict showed a Cronbach’s Alpha of 0.797 and an AVE of 0.708, indicating robust internal consistency and a significant portion of variance captured by the construct^126^. Similarly, life-work conflict exhibited high reliability with a Cronbach’s Alpha of 0.913 and an AVE of 0.920, signifying strong construct validity.

**Table 2:** Scale Reliability and Validity.

Construct	Items	Mean	St. Dev.	Factor Loading	Cronbach’s Alpha	CR(rho_a)	CR(rho_c)	AVE
Work-life Conflict	WLC1	1.963	0.964	0.715	0.797	0.893	0.878	0.708
	WLC2	2.002	0.949	0.923				
	WLC3	1.928	0.917	0.872				
Life-work Conflict	LWC1	1.591	0.726	0.960	0.913	0.913	0.958	0.920
	LWC2	1.566	0.703	0.958				
Supervisory Coaching	COA1	3.861	0.707	0.877	0.873	0.874	0.922	0.797
	COA2	3.797	0.730	0.900				
	COA3	3.691	0.732	0.902				
Women’s Proportion	WOM1	2.977	1.401	1.000	–	–	–	–
Household Size	SIZ1	2.607	1.211	1.000	–	–	–	–
Supervisor Gender	SGE1	0.302	0.441	1.000	–	–	–	–
Engagement	EGM1	3.453	0.762	0.877	0.810	0.815	0.888	0.725
	EGM2	3.559	0.812	0.881				
	EGM3	3.641	0.819	0.794				
Subjective Well-being	SUB1	3.986	1.152	0.912	0.865	0.875	0.917	0.787
	SUB2	4.065	1.159	0.901				
	SUB3	3.423	1.284	0.847				
Sexual Harassment	SHA1	0.090	0.370	1.000	–	–	–	–
Violence	VLC1	0.012	0.110	1.000	–	–	–	–

The Fornell-Larcker criterion results in Table 3 further confirmed the discriminant validity of the constructs. The square root of AVE for each construct was greater than its highest correlation with any other construct, indicating that each construct uniquely captures the variance of its indicators^126^. For example, the square root of AVE for Work-life Conflict (0.841) was higher than its highest correlation with any other construct, supporting discriminant validity.

**Table 3:** Fornell-Larcker Scale Results.

Construct	1	2	3	4	5
1. Work-life Conflict	0.841				
2. Life-work Conflict	0.635	0.959			
3. Supervisory Coaching	-0.128	-0.118	0.893		
4. Engagement	-0.115	-0.112	0.343	0.852	
5. Subjective well-being	-0.118	-0.050	0.259	0.556	0.887

Diagonal elements are the square root of AVE. Only multi-item reflective constructs are included here (e.g., work-life conflict, life-work conflict, supervisory coaching, engagement, subjective well-being). Single-item or categorical variables (women’s proportion, supervisor gender, household size, sexual harassment, and violence) are excluded since AVE and CR cannot be calculated for them.

Additionally, the Heterotrait-Monotrait (HTMT) ratio of correlations in Table 4 was employed as another criterion for assessing discriminant validity^127^. The HTMT values were below the threshold of 0.85 for all construct pairs, further confirming discriminant validity. For instance, the HTMT value between work-life conflict and life-work conflict was 0.749, well below the 0.85 threshold.

**Table 4:** HTMT Matrix.

Construct	1	2	3	4	5
1. Work-life Conflict					
2. Life-work Conflict	0.749				
3. Supervisory Coaching	0.143	0.132			
4. Engagement	0.134	0.131	0.407		
5. Subjective well-being	0.132	0.074	0.295	0.657	

HTMT values are reported only for reflective constructs with multiple items. Variables measured with a single item or categorical format are not applicable and therefore omitted.

### Structural model

The structural model of this study was rigorously analyzed using PLS-SEM with 5000 resampling bootstraps to validate the proposed hypotheses. The results, as detailed in Table 5, indicate the strength and significance of relationships between the constructs.

**Table 5:** SEM Results.

H	Predictor	Outcome	β	t	*p*	Result
H1a	Work-life Conflict	Engagement	-0.043	5.851	0.000	Supported
H1b	Work-life Conflict	Subjective Well-being	-0.124	17.199	0.000	Supported
H2a	Life-work Conflict	Engagement	-0.045	6.174	0.000	Supported
H2b	Life-work Conflict	Subjective Well-being	0.056	7.683	0.000	Not Supported (Significant)
H3a	Supervisory Coaching	Engagement	0.332	60.098	0.000	Supported
H3b	Supervisory Coaching	Subjective Well-being	0.246	45.174	0.000	Supported
H4a	Women’s proportion	Engagement	0.006	0.916	0.360	Not Supported
H4b	Women’s proportion	Subjective Well-being	0.015	2.353	0.019	Supported
H5a	Household Size	Life-work Conflict	0.038	6.807	0.000	Supported
H5b	Household Size	Subjective Well-being	0.046	8.609	0.000	Supported
H6a	Supervisor Gender	Supervisory Coaching	0.066	5.139	0.000	Supported
H6b	Supervisor Gender	Engagement	-0.027	1.966	0.049	Supported
H6c	Supervisor Gender	Subjective Well-being	0.009	0.606	0.544	Not Supported
H7	Sexual Harassment	Subjective Well-being	-0.019	3.223	0.001	Supported
H8	Violence	Subjective Well-being	-0.051	1.010	0.313	Not Supported

This study also examined explanatory power (*R²*), effect sizes (*f²*), predictive relevance (*Q²*), and model fit (SRMR), following recommendations for comprehensive model evaluation. The *R²* values indicated that the model explained 12.4% of the variance in engagement and 7.9% of the variance in subjective well-being, while life-work conflict and supervisory coaching had negligible explained variance. Effect size (*f²*) analysis revealed that supervisory coaching had a medium effect on engagement (*f²* = 0.123) and a small-to-medium effect on subjective well-being (*f²* = 0.064). Other predictors showed very small effect sizes. Predictive relevance (*Q²*) values were 0.073 for engagement and 0.055 for subjective well-being, both exceeding zero, confirming acceptable predictive accuracy (Table 6). Regarding model fit, the SRMR was 0.037 for the saturated model and 0.130 for the estimated model. The saturated SRMR value was well below the threshold of 0.08, suggesting a good overall model fit^128^.

**Table 6:** Effect Sizes (*f*^*2*^).

Predictor Construct	Outcome Construct	f² Effect Size
Work-life Conflict	Engagement	0.001
Work-life Conflict	Subjective Well-being	0.010
Life-work Conflict	Engagement	0.001
Life-work Conflict	Subjective Well-being	0.002
Supervisory Coaching	Engagement	0.123
Supervisory Coaching	Subjective Well-being	0.064
Women’s Proportion	Engagement	0.000
Women’s Proportion	Subjective Well-being	0.000
Household Size	Life-work Conflict	0.001
Household Size	Subjective Well-being	0.002
Supervisor Gender	Supervisory Coaching	0.001
Supervisor Gender	Engagement	0.000
Supervisor Gender	Subjective Well-being	0.000
Sexual Harassment	Subjective Well-being	0.000
Violence	Subjective Well-being	0.000

### Mediation analysis

To further examine the relationships among the variables in the research model, mediation effects were analyzed to determine whether certain workplace factors operate indirectly through intermediate constructs. As shown in Table 7, household size exerted a significant indirect effect on both engagement and subjective well-being through life-work conflict. Specifically, larger household size increased life-work conflict, which subsequently reduced engagement but unexpectedly enhanced subjective well-being. This result aligns with COR theory, which suggests that while increased family demands may drain resources needed for work, they can also provide emotional support that strengthens overall well-being^41^. Similarly, supervisor gender indirectly affected engagement and subjective well-being through supervisory coaching. Male and female supervisors demonstrated different coaching tendencies, and this variation shaped employee outcomes. The significant mediation pathways indicate that coaching behaviors are a critical mechanism linking supervisor gender with employee engagement and well-being. These findings highlight the importance of examining indirect effects to capture the complex processes underlying workplace dynamics.

**Table 7:** Mediation Effects.

Path	β	t	*p*
Household Size ↴ Life-work Conflict ↴ Engagement	-0.002	4.595	0.000
Household Size ↴ Life-work Conflict ↴ Subjective Wellbeing	0.002	5.128	0.000
Supervisor Gender ↴ Supervisory Coaching ↴ Engagement	0.022	5.112	0.000
Supervisor Gender ↴ Supervisory Coaching ↴ Subjective Wellbeing	0.016	5.094	0.000

## Discussion

In this study, the complex interplay between workplace factors and employee outcomes was dissected, revealing nuanced insights that contribute significantly to our understanding of workplace dynamics.

The negative impact of work-life conflict on both employee engagement and subjective well-being confirms the considerable strain that competing demands between personal and professional life place on individuals. The findings corroborate the extensive literature that has consistently highlighted the deleterious effects of work-life conflict on employee morale and mental health^43,57,61,129,130^. Like some Western studies that emphasize flexible work as a buffer^131^, this study also highlights the negative consequences of conflict within the rigid work culture of South Korea, indicating a context-specific amplification of stressors. This research supports the notion that mitigating these conflicts is crucial for maintaining workforce vitality and productivity, aligning with previous studies which advocate for organizational strategies that address work-life balance to enhance employee satisfaction and organizational commitment^58,62,66,132^. Organizations can mitigate work-life conflict by implementing flexible scheduling, reducing excessive overtime, and promoting work-from-home arrangements to preserve employee energy and reduce stress.

Conversely, life-work conflict showed a divergent impact on engagement and subjective well-being. While it diminished engagement, it surprisingly had a positive coefficient for subjective well-being. This result can be interpreted within the framework of the COR theory, which posits that personal and social resources—such as family responsibilities and caregiving roles—may provide individuals with emotional fulfillment and a sense of purpose that enhance overall well-being^41^. Although life-work conflict consumes time and energy that reduce engagement, these same personal responsibilities may serve as resource-providing elements, offsetting stress and supporting subjective well-being. The JD-R model further explains this duality by recognizing that certain demands can simultaneously function as resources, offering meaning and satisfaction despite their costs^39,133,134^. This complexity highlights the dual-edged nature of life-work balance, suggesting that personal life responsibilities can act as both constraints and resources depending on context, a phenomenon partially supported by Halbesleben^68^ and deserving deeper exploration in future studies. While previous studies generally report negative outcomes of life-work conflict^2,10,65,66,135,136^, the positive association with well-being observed here diverges from dominant findings. This suggests that cultural values surrounding family obligations in collectivist societies may transform life demands into sources of meaning and resilience. Moreover, cultural and gendered expectations provide further explanation for this divergence. In collectivist societies such as South Korea, family responsibilities are often framed not solely as obligations but as socially valued contributions that reinforce identity and belonging. Within this context, caregiving roles—particularly for women—are tied to cultural notions of fulfillment and moral duty, which may enhance subjective well-being even when they interfere with work. This suggests that the meaning attached to personal obligations is culturally contingent, and that family-related demands in collectivist societies can be reinterpreted as sources of resilience and emotional gain rather than purely as stressors. Managers can reframe family responsibilities as resources by offering family-friendly benefits such as childcare support, caregiving leave, and recognition programs that validate personal obligations as part of holistic employee well-being.

The role of supervisory coaching was found to be profoundly positive, significantly enhancing both engagement and subjective well-being. This supports earlier findings that coaching behaviors enhance job attitudes^70,71^. This underscores the importance of quality leadership and mentorship in the workplace, consistent with literature that emphasizes how supportive supervisory behaviors foster job satisfaction, empowerment, and personal development^16,69,137,138^. Within the JD-R model, supervisory coaching functions as a critical job resource that not only energizes employees but also buffers against stress, thereby strengthening well-being. The results emphasize the transformative potential of effective leadership and suggest that enhancing supervisory skills should be a strategic focus for organizations aiming to uplift workforce morale and productivity. HR departments should prioritize supervisory training programs that emphasize coaching, mentoring, and emotional intelligence to ensure leaders are equipped to foster both engagement and resilience among employees.

The influence of women’s proportion in the workplace presented mixed results: while its effect on engagement was not supported, it showed a positive impact on subjective well-being. This suggests that while the presence of women in significant numbers may not directly boost engagement, it contributes to a psychologically supportive and inclusive work environment that enhances mental health and satisfaction. Prior studies have linked gender diversity to improved innovation and organizational effectiveness^50,73,139^. The present results extend this evidence by showing that in collectivist cultures, women’s representation may not directly stimulate engagement but strongly contributes to well-being through inclusiveness and social support. From a COR perspective, gender diversity may serve as a resource-providing element that buffers against workplace stressors, aligning with research that highlights how inclusiveness strengthens organizational health and employee well-being^50,73^. Organizations can strengthen the positive effects of gender diversity by ensuring equitable promotion opportunities, implementing mentorship networks for women, and fostering inclusive team dynamics that enhance well-being.

Household size’s effect on life-work conflict and subjective well-being was also supported, underscoring the importance of personal life dimensions in shaping workplace experiences. Larger household sizes were associated with increased life-work conflict, reflecting greater domestic responsibilities that compete with work demands. However, they also contributed positively to subjective well-being, suggesting that while larger families may create logistical and time-related challenges, they simultaneously provide emotional and social resources that enhance life satisfaction^91,95,140,141^. While earlier research highlights household responsibilities as stressors^92,142,143^, the current study reveals their simultaneous role as social resources. This aligns with findings that family support mitigates occupational stress^144,145^, but differs by showing both costs and benefits operating together in the same model. From the perspective of COR theory, these findings highlight that family support functions as a compensatory resource that offsets stress and fosters resilience, even when it coexists with higher conflict levels. Employers could design family-supportive policies, such as dependent-care subsidies and flexible leave systems, to balance increased domestic responsibilities while reinforcing the social resources families provide.

The examination of supervisor gender revealed that it significantly influences supervisory coaching and engagement but not subjective well-being. This indicates that while the gender of a supervisor can shape coaching effectiveness and relational dynamics that foster engagement, its direct impact on subjective well-being may be limited. One possible explanation lies in gender role theory, which suggests that female supervisors are often perceived as more communal and supportive, aligning with behaviors that enhance engagement, while male supervisors may be associated with task-oriented approaches^21,146^. These gendered expectations can influence how employees respond to coaching behaviors and engagement outcomes. However, subjective well-being is a broader construct, often shaped by multiple organizational and personal resources, which may dilute the influence of supervisor gender alone. Another perspective stems from the perceived leadership competence framework, which argues that leadership effectiveness is judged through the lens of gender stereotypes and congruence with expected roles^147^. As such, differences in engagement may reflect immediate perceptions of leadership style, while well-being outcomes remain more resilient to gender-based influences. Leadership development initiatives should incorporate awareness of gendered communication styles, helping supervisors of all genders adopt inclusive approaches that sustain engagement without bias.

Lastly, the roles of sexual harassment and violence in affecting subjective well-being were also explored. While sexual harassment had a predictably negative impact, the influence of workplace violence was not supported in the findings. One possible explanation is that violence may be underreported due to cultural stigma or fear of retaliation, particularly in hierarchical workplace contexts such as South Korea, where employees may hesitate to disclose such experiences. Another interpretation lies in the COR, which suggests that resource depletion is context-dependent; employees exposed to isolated incidents of violence may rely on social or organizational support to buffer negative outcomes, thereby diluting its measurable impact on well-being^41,113,148^. Furthermore, research indicates that while harassment is often persistent and relational, workplace violence can be episodic and less chronic, which may limit its cumulative effect on subjective well-being^149^. Methodological factors, such as the use of single-item measures, may also constrain the sensitivity of detecting these effects in large-scale surveys. Together, these points suggest that the non-significant results may reflect a combination of cultural, theoretical, and methodological factors that warrant deeper exploration in future research. Strong anti-harassment policies, confidential reporting mechanisms, and proactive violence-prevention training are essential strategies to enhance psychological safety and minimize negative well-being outcomes.

The findings of this study should be interpreted in the context of South Korea’s cultural and organizational environment. South Korea is characterized by strong collectivist values^150^, hierarchical workplace structures^151^, and high expectations for employee commitment, which can intensify work-life conflict and shape its consequences. In such settings, long working hours and organizational loyalty are often prioritized over personal life, making the boundary between work and family domains particularly rigid. At the same time, family and household responsibilities remain deeply influential, reinforcing the dual pressures employees face. These cultural and structural characteristics help explain why work-life and life-work conflicts emerge so prominently and why resources such as supervisory coaching and gender diversity take on heightened importance in shaping engagement and well-being. Therefore, while the results provide valuable insights, their generalizability should be considered in light of the Korean context, and cross-cultural studies are encouraged to test the robustness of these findings.

The novelty of this study lies in its multidimensional exploration of workplace dynamics, integrating both organizational and personal factors within the frameworks of the JD-R model, COR theory, and SET. Unlike prior research that often treated these constructs in isolation, this study simultaneously examined work-life conflict, life-work conflict, supervisory coaching, women’s proportion, household size, supervisor gender, sexual harassment, and violence to provide a holistic understanding of employee engagement and well-being. Theoretically, the study contributes by showing how certain stressors, such as life-work conflict, can paradoxically function as resources within the COR framework, and how supervisory coaching serves as a critical job resource in the JD-R model. Furthermore, the findings highlight gender-related dynamics, including the nuanced role of supervisor gender and women’s workplace proportion, extending the application of gender role theory and leadership competence frameworks. By contextualizing results within South Korea’s collectivist and hierarchical work culture, the study enriches cross-cultural organizational research and underscores the importance of tailoring theoretical models to cultural settings.

### Theoretical contribution

This study enhances the understanding of how distinct workplace factors influence employee engagement and subjective well-being by integrating core organizational theories in a novel empirical framework. Unlike previous research, which often considers work-life dynamics as monolithic phenomena, this research delineates between work-life conflict and life-work conflict, providing a nuanced view of their differential impacts.

Firstly, the study contributes to the JD-R model by dissecting the dual nature of work-life and life-work conflicts as separate constructs. Previous studies often aggregate these conflicts, potentially obscuring unique effects on employee outcomes. This research confirms that work-life conflict negatively impacts both engagement and well-being^39^, consistent with earlier findings. However, it uniquely demonstrates that life-work conflict, while also diminishing engagement, may paradoxically enhance subjective well-being. This suggests that personal responsibilities, typically viewed solely as distractions or burdens in the workplace, can sometimes provide a sense of accomplishment or fulfillment that contributes positively to well-being. This finding challenges the traditional narrative found in studies by Halbesleben^68^ and introduces a complex dynamic where not all conflicts are detrimental in all outcomes.

Secondly, the study extends the COR by exploring how supervisor gender and household size serve as resources or stressors that influence the conservation or depletion of personal and professional resources. While previous research has touched upon the role of supervisor gender^21^, this study is one of the few to empirically test its impact on supervisory coaching and, by extension, on engagement and well-being. The findings indicate that supervisor gender does not universally affect well-being, suggesting that other interpersonal or organizational factors may mediate this relationship. Additionally, the positive implications of supervisory coaching on both engagement and well-being reinforce the importance of leadership quality in resource provision. This aligns with SET^56^, where supportive supervisory behaviors are reciprocated with higher employee engagement and improved well-being, thus creating a beneficial cycle of resource enrichment.

Beyond extending JD-R, COR, and SET, this study also advances cross-cultural management theory by confirming and revising prior findings in a new national context. Specifically, the unexpected positive association between life-work conflict and subjective well-being illustrates how collectivist cultural values in South Korea reframe personal obligations as meaningful resources rather than solely as stressors. This finding challenges Western-centric assumptions that all role conflicts are uniformly detrimental and highlights the importance of cultural and gendered contexts in shaping the work–life–well-being nexus. Moreover, by integrating gendered workplace structures—such as supervisor gender and women’s proportion—the study situates well-being within equity and inclusivity debates, offering a nuanced framework that future research can apply across diverse cultural settings.

Scholars should consider these findings as a call to further investigate the conditions under which life-work conflict might yield positive subjective well-being and to explore the mediating factors that differentiate the impacts of supervisor gender. This study suggests a more segmented approach to examining work-life dynamics, advocating for research designs that can isolate and analyze the effects of these dynamics more precisely. Future research could benefit from a longitudinal approach to better understand the causal relationships and potentially changing impacts of these workplace factors over time. Moreover, incorporating cross-cultural comparisons could elucidate how cultural contexts influence the perception and effects of work-life and life-work conflicts.

### Practical implication

The findings of this study provide clear and differentiated guidance for managers, HR specialists, and policy makers.

For managers, the evidence that work-life and life-work conflict affect engagement and well-being differently suggests the importance of offering flexible work arrangements, such as remote work or adjustable schedules, to reduce stress and improve engagement. Supervisory coaching also emerged as a powerful positive factor. To implement this effectively, organizations can establish structured coaching programs that include regular one-on-one sessions, feedback workshops, and mentoring systems, supported by measurable goals and performance tracking.

For HR specialists, the results highlight the need to embed supervisory coaching into leadership development programs, with a strong emphasis on emotional intelligence and supportive practices. This could involve mandatory training modules on coaching skills, role-playing exercises to strengthen empathy, and certification systems to ensure consistent quality. Gender diversity was found to enhance well-being, indicating that HR departments should implement inclusive recruitment and promotion strategies, mentorship networks, and policies that actively foster equitable representation across organizational levels.

For policy makers, the study underlines the vulnerability of temporary employees and women who experience higher work-life conflict. Public policy could support these groups through regulatory frameworks that encourage organizational investment in stress management programs, mandatory health screenings, and workplace counseling services. Additionally, to address sexual harassment, policymakers should strengthen reporting mechanisms by mandating anonymous reporting platforms, protecting whistleblowers, and promoting awareness campaigns that shift organizational cultures toward zero tolerance. Such interventions would not only protect employee health but also foster safe, equitable, and sustainable workplaces.

## Limitation and future research directions

One of the limitations of this study is its reliance on self-reported data, which may introduce bias or inaccuracies in measuring workplace conflicts and their psychological impacts. Future research could benefit from incorporating objective data, such as organizational records of employee performance and health outcomes, to validate and extend the findings. A second limitation is that some constructs, such as women’s proportion, household size, and supervisor gender, were measured using single-item indicators due to the nature of secondary data. While these items provide meaningful insights, they inherently limit reliability compared to multi-item latent constructs. Future studies should aim to employ multi-item scales or triangulate these measures with qualitative or administrative data to strengthen validity. Additionally, while this study provides initial insights into the differential impacts of work-life dynamics across genders and employment types, further research is needed to explore these dynamics in a variety of cultural and economic contexts to enhance the generalizability of the findings. Researchers are encouraged to replicate this study in different cultural settings and to consider longitudinal designs that could more clearly capture the causal relationships and potential long-term effects of the examined factors on employee well-being and engagement.

## Electronic Supplementary Material

Below is the link to the electronic supplementary material.

Supplementary Material 1

## Data Availability

The dataset is publicly available and can be freely accessed and downloaded from the official KOSHA data portal: https://oshri.kosha.or.kr/eoshri/resources/KWCSDownload.do.
