# Supplementary material for: Work–life conflict, coaching, and workplace harassment as determinants of employee well-being
Source: Sci Rep. 2026 Apr 30;16:20073. doi: 10.1038/s41598-026-51240-4 (PMC13324475; doi:10.1038/s41598-026-51240-4)
Supplement: Supplementary file 1 — Supplementary Material 1 [file 41598_2026_51240_MOESM1_ESM.docx]

# Appendix

Table A1. List of Constructs and Items

| Construct | Items | Description | Related Work |
| --- | --- | --- | --- |
| Work-Life  Conflict | WLC1 | I keep worrying about work even when I'm not working. | [Boogar et al. (2018)](#_ENREF_4)  ;[Han and Kwak (2022)](#_ENREF_10) |
|  | WLC2 | I am so tired after work that I can't complete the housework I need to do. |  |
|  | WLC3 | I cannot dedicate as much time to my family as I would like because of work. |  |
| Life-Work  Conflict | LWC1 | It is difficult to concentrate on work because of housework. | [Boogar et al. (2018)](#_ENREF_4)  ;[Han and Kwak (2022)](#_ENREF_10) |
|  | LWC2 | I can't spend enough time on my work because of housework. |  |
| Supervisory  Coaching |  | How is your immediate supervisor in the following aspects? Please select the appropriate response for each item. | [Schaufeli and Bakker (2004)](#_ENREF_14) |
|  | COA1 | It is helpful in handling the work. |  |
|  | COA2 | Useful advice (feedback) is given about the work. |  |
|  | COA3 | I am encouraged and helped to develop. |  |
| Women’s  Workplace  Proportion | WOM1 | What is the approximate percentage of women among the employees at your current place of employment (company)? Please select the closest option:  1. None or almost none 2. less than half 3. about half 4. more than half 5. all or almost all | [Elwér et al. (2014)](#_ENREF_8) |
| Household  Size | SIZ1 | Household Size | [Carr et al. (2019)](#_ENREF_6) |
| Supervisor  Gender | SGE1 | Is your immediate supervisor (direct superior) who instructs and manages your work male or female? | [Paustian‐Underdahl et al. (2017)](#_ENREF_12)  ;[Vial et al. (2018)](#_ENREF_16)  ;[Moore et al. (2005)](#_ENREF_11) |
| Engagement |  | How often do you experience the following emotions while working? | [Trépanier et al. (2014)](#_ENREF_15)  ;[Birkeland and Buch (2015)](#_ENREF_3) |
|  | EGM1 | I feel full of energy when I work. |  |
|  | EGM2 | I am passionate in my work. |  |
|  | EGM3 | Time flies when I work. |  |
| Subjective  Well-being |  | In the following questions, please indicate how often you have experienced these feelings in the past two weeks. | [Agarwal et al. (2019)](#_ENREF_2)  ;[Saaranen et al. (2007)](#_ENREF_13)  ;[Butt et al. (2020)](#_ENREF_5) |
|  | SUB1 | I feel calm and comfortable. |  |
|  | SUB2 | I am active and energetic. |  |
|  | SUB3 | My daily life is full of interesting things. |  |
| Sexual  Harassment | SHA1 | In the past month, have you experienced any of the following while performing your duties?  A. Unwanted sexual attention, B. Sexual harassment | [Glomb et al. (1999)](#_ENREF_9)  ;[Worke et al. (2023)](#_ENREF_17) |
| Violence | VLC1 | In the past month, have you experienced any of the following while performing your duties?  A. Verbal abuse, B. Threats, C. Offensive behavior, D. Physical violence, E. Bullying/Harassment | [Chang et al. (2012)](#_ENREF_7)  ;[Adinyira et al. (2020)](#_ENREF_1) |

# Reference

Adinyira, E., Manu, P., Agyekum, K., Mahamadu, A.-M., & Olomolaiye, P. O. (2020). Violent behaviour on construction sites: structural equation modelling of its impact on unsafe behaviour using partial least squares. *Engineering, Construction and Architectural Management*, *27*(10), 3363-3393.

Agarwal, S., Garg, P., & Rastogi, R. (2019). Testing the reciprocal relationship between quality of work life and subjective well-being: a path analysis model. *International Journal of Project Organisation and Management*, *11*(2), 140-153.

Birkeland, I. K., & Buch, R. (2015). The dualistic model of passion for work: Discriminate and predictive validity with work engagement and workaholism. *Motivation and Emotion*, *39*(3), 392-408.

Boogar, I. R., Talepasand, S., & Mashhadi, B. B. (2018). Quality of Nursing Work Life, Work-Family Conflict, and Self-Regulation: A Structural Equation Modeling. *International Journal of Occupational Hygiene*, *10*(3), 114-123.

Butt, T. H., Abid, G., Arya, B., & Farooqi, S. (2020). Employee energy and subjective well-being: a moderated mediation model. *The Service Industries Journal*, *40*(1-2), 133-157.

Carr, S., Haar, J., Hodgetts, D., Arrowsmith, J., Parker, J., Young-Hauser, A., Alefaio-Tuglia, S., & Jones, H. (2019). An employee’s living wage and their quality of work life: How important are household size and household income? *Journal of Sustainability Research*.

Chang, C. H., Eatough, E. M., Spector, P. E., & Kessler, S. R. (2012). Violence‐prevention climate, exposure to violence and aggression, and prevention behavior: A mediation model. *Journal of organizational behavior*, *33*(5), 657-677.

Elwér, S., Johansson, K., & Hammarström, A. (2014). Workplace gender composition and psychological distress: the importance of the psychosocial work environment. *BMC Public Health*, *14*, 1-9.

Glomb, T. M., Munson, L. J., Hulin, C. L., Bergman, M. E., & Drasgow, F. (1999). Structural equation models of sexual harassment: Longitudinal explorations and cross-sectional generalizations. *Journal of Applied Psychology*, *84*(1), 14.

Han, S., & Kwak, S. (2022). The effect of sleep disturbance on the association between work–family conflict and burnout in nurses: a cross-sectional study from South Korea. *BMC Nursing*, *21*(1), 354. <https://doi.org/10.1186/s12912-022-01114-7>

Moore, S., Grunberg, L., & Greenberg, E. (2005). Are female supervisors good for employee job experiences, health, and wellbeing? *Women in Management Review*, *20*(2), 86-95.

Paustian‐Underdahl, S. C., King, E. B., Rogelberg, S. G., Kulich, C., & Gentry, W. A. (2017). Perceptions of supervisor support: Resolving paradoxical patterns across gender and race. *Journal of Occupational and Organizational psychology*, *90*(3), 436-457.

Saaranen, T., Tossavainen, K., Turunen, H., Kiviniemi, V., & Vertio, H. (2007). Occupational well-being of school staff members: a structural equation model. *Health education research*, *22*(2), 248-260.

Schaufeli, W. B., & Bakker, A. B. (2004). Job demands, job resources, and their relationship with burnout and engagement: A multi‐sample study. *Journal of Organizational Behavior: The International Journal of Industrial, Occupational and Organizational Psychology and Behavior*, *25*(3), 293-315.

Trépanier, S.-G., Fernet, C., Austin, S., Forest, J., & Vallerand, R. J. (2014). Linking job demands and resources to burnout and work engagement: Does passion underlie these differential relationships? *Motivation and Emotion*, *38*, 353-366.

Vial, A. C., Brescoll, V. L., Napier, J. L., Dovidio, J. F., & Tyler, T. R. (2018). Differential support for female supervisors among men and women. *Journal of Applied Psychology*, *103*(2), 215.

Worke, M. D., Koricha, Z. B., & Debelew, G. T. (2023). Consequences of exposure to sexual harassment among women working in hospitality workplaces in Bahir Dar City, Ethiopia: a structural equation model. *Archives of Public Health*, *81*(1), 1-16.
